# Discovery of cold-resistance genes in *Vitis amurensis* using bud-based quantitative trait locus mapping and RNA-seq

**DOI:** 10.1186/s12864-022-08788-y

**Published:** 2022-08-03

**Authors:** Xiaolele Ma, Fangyuan Zhao, Kai Su, Hong Lin, Yinshan Guo

**Affiliations:** 1 College of Horticulture, Shenyang Agricultural University, Shenyang, 110866 People’s Republic of China; 2 National & Local Joint Engineering Research Center of Northern Horticultural Facilities Design and Application Technology (Liaoning), Shenyang, 110866 People’s Republic of China; 3 College of Horticulture Science and Technology, Hebei Normal University of Science and Technology, Qinhuangdao, 066004 People’s Republic of China; 4 Hebei Key Laboratory of Horticultural Germplasm Excavation and Innovative Utilization, Qinhuangdao, 066004 People’s Republic of China

**Keywords:** *Vitus amurensis*, Grape bud, Cold resistance breeding, Transcriptome analysis, QTL mapping

## Abstract

**Background:**

In cold regions, low temperature is the main limiting factor affecting grape production. As an important breeding resource, *V. amurensis* Rupr. has played a crucial role in the discovery of genes which confer cold resistance in grapes. Thus far, many cold-resistance genes have been reported based on the study of *V. amurensis*. In order to identify more candidate genes related to cold resistance in *V. amurensis*, QTL mapping and RNA-seq was conducted based on the hybrid population and different cold-resistance cultivars in this study.

**Results:**

In this study, highly cold-resistant grape cultivar ‘Shuangyou’ (SY) which belongs to *V. amurensis,* and cold-sensitive cultivar ‘Red Globe’ (RG) which belongs to *Vitis vinifera* L*.* were used to identify cold resistance genes. Cold-resistance quantitative trait locus (QTL) mapping was performed based on genetic population construction through interspecific crossing of ‘Shuangyou’ and ‘Red Globe’. Additionally, transcriptome analysis was conducted for the dormant buds of these two cultivars at different periods. Based on transcriptome analysis and QTL mapping, many new structural genes and transcription factors which relate to *V. amurensis* cold resistance were discovered, including CORs (*VaCOR413IM*), GSTs (*VaGST*-*APIC*, *VaGST*-*PARB*, *VaGSTF9* and *VaGSTF13*), ARFs (*VaIAA27* and *VaSAUR71*), ERFs (*VaAIL1*), MYBs (*VaMYBR2*, *VaMYBLL* and *VaMYB3R-1*) and bHLHs (*VaICE1* and *VabHLH30*).

**Conclusions:**

This discovery of candidate cold-resistance genes will provide an important theoretical reference for grape cold-resistance mechanisms, research, and cold-resistant grape cultivar breeding in the future.

**Supplementary Information:**

The online version contains supplementary material available at 10.1186/s12864-022-08788-y.

## Introduction

Grape is one of the most important fruit species in the world and it is also the main cultivated fruit crop in China. China is located in a region with a typical continental monsoon climate. The grape cultivars in most grape production regions are sensitive to cold. The grapevines should be buried in soil when the temperature falls below -15 ℃ to survive the cold environment in winter. This not only increases the production cost of the vineyard, but also causes a series of problems such as tree body damage, soil erosion, and soil horizon destruction that considerably limited the development of grape production. Many grape species such as *Vitis riparia* Michx, *Vitis rupestris* Scheele and *Vitis labrusca* L. indigenous to North America, *Vitis amurensis* Rupr. and *Vitis yeshanensis J.X.Chen* from Eastern Asia possess strong resistance to cold conditions [1]. The cold resistance of *V. amurensis* is the highest among resistant species, and its cane can survive in temperatures of -40 to -50 ℃. Therefore, it is an excellent germplasm resource for cold-resistance breeding as seed-parent.

Thus far, there have been many reports related to genetic and transcriptional regulation mechanisms of grape cold resistance based on *V. amurensis*. Interaction of *VaPAT1* and *VaIDD3* in *V. amurensis* could enhance the expression of *VaLOX*, which plays a crucial role in jasmonic acid synthesis and improves cold resistance [2]. Genome-wide association analysis shows that phosphoglycerate kinase gene (PGK) in *V. amurensis* significantly influences cold resistance [3]. A number of loci and candidate genes related to grapevine cane cold-hardiness was discovered based on the hybrid population constructed using ‘Cabernet Sauvignon’ and ‘Zuoyouhong’ [4]. Ethylene response factor VaERF092 improves cold resistance in *V. amurensis* by binding to the GCC-box element in the promoter region of *VaWRKY33,* thus promoting its expression [5]. Furthermore, candidate cold-resistance genes identified based on *V. amurensis* were reported, including *VaDof17d* [6], *VaAQUILO* [7], *VaERD15* [8], and *VaWRKY12* [9]. Candidate cold-resistance genes in *V. vinifera* were discovered, including *VvNAC17* [10], *VV*-*circATS1* [11], and *VvBAP* [12] and the function of these genes has been preliminarily verified in *Arabidopsis thaliana*. In summary, *V. amurensis* plays an important role in the discovery of cold-resistance genes. However, plants exposed to chilling (0 – 15 ℃) and freezing (< 0 ℃) stress show differing gene expression patterns [13, 14], and most previous studies have focused on chilling stress.

In this study, based on the hybrid population created through interspecific crossing of cold-resistance cultivar ‘Shuangyou’ (SY) and cold-sensitive cultivar ‘Red Globe’ (RG) and the high-density genetic linkage map [15], we conducted QTL mapping for cold resistance of grape dormant buds firstly and then transcriptome analysis was conducted for ‘SY’ and ‘RG’ buds at different dormant stages. Finally, candidate genes related to cold resistance of grape buds were screened by QTL mapping and RNA-seq.

## Results

### Identification of cold resistance

**Fig. 1:**
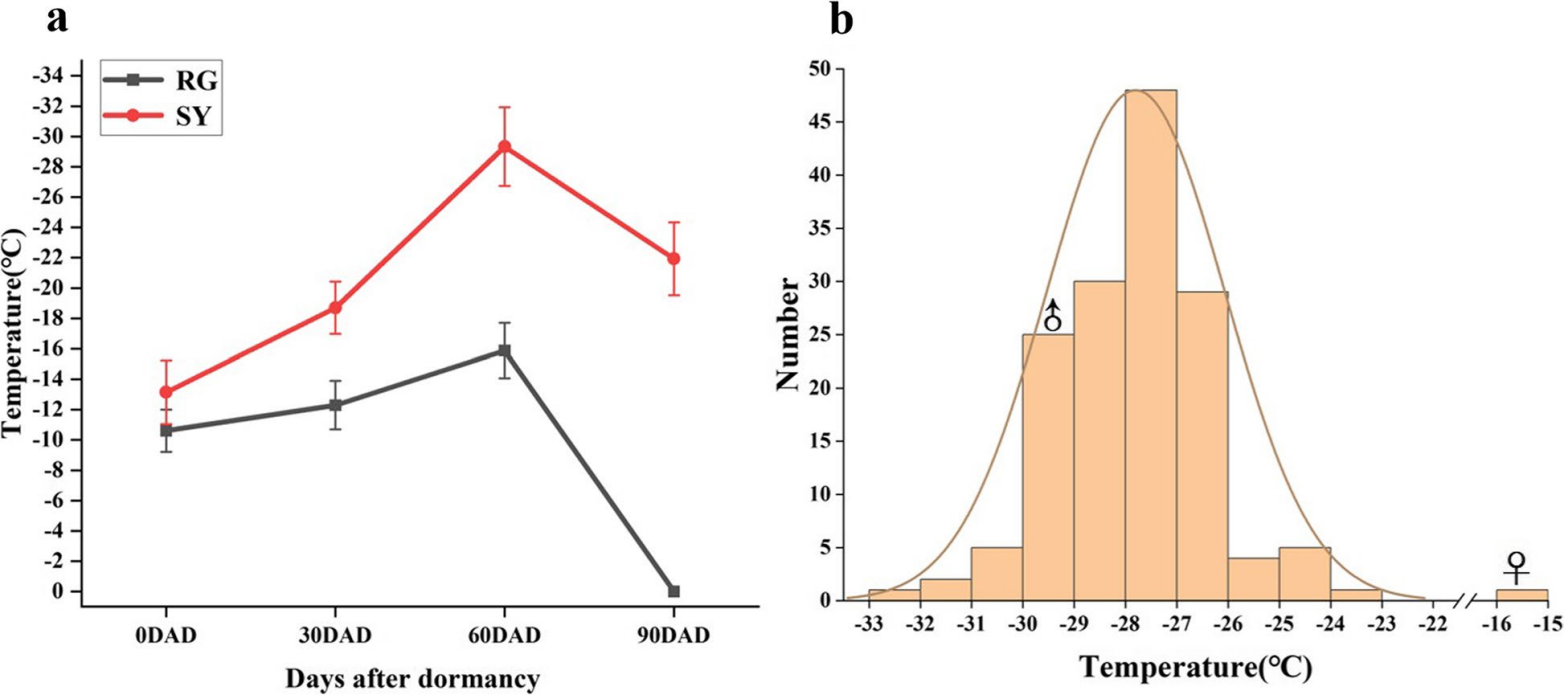
Characterization of cold resistance for grape cultivar ‘Shuangyou’ (SY), ‘Red Globe’(RG) and their hybrid offspring buds. **a** Characterization of cold resistance in ‘SY’ and ‘RG’ buds at different dormant stage. X axis represents days after Oct 20, 2021 which was defined as the beginning of bud dormancy. Error bars represent the standard deviation of three biological replicates. **b** Characterization of cold resistance in hybrid offspring buds.“**♂**” represents the temperature interval of male parent and ‘SY’ and “**♀**” represents the temperature interval of female parent ‘RG’

Cold resistance identification of ‘SY’ and ‘RG’ buds at four different dormant stages was evaluated by Differential Thermal Analysis (DTA) analysis. The cold resistance of the buds of both cultivars increased with time when measured over 60 days after dormancy (DAD) began. At 60DAD, the cold resistance of the grape cultivars ‘SY’ and ‘RG’ reached the peak value of the heat release curve, and the cold resistance of ‘SY’ was significantly higher than ‘RG’ at 90 days after dormancy. The cold resistance of ‘RG’ was significantly reduced (Fig. 1a). Furthermore, 149 hybrid progenies of ‘SY’ and ‘RG’ were identified for cold resistance after dormancy, and the results showed continuous variation (Fig. 1b). These results indicated that the cold resistance of grape cultivar ‘SY’ was significantly higher than that of grape variety ‘RG’ and the cold resistance in grape was a typical quantitative trait controlled by multiple genes. Finally, eight individuals which showed greater cold resistance were selected.

### Transcriptome and differentially expressed gene analysis

**Fig. 2:**
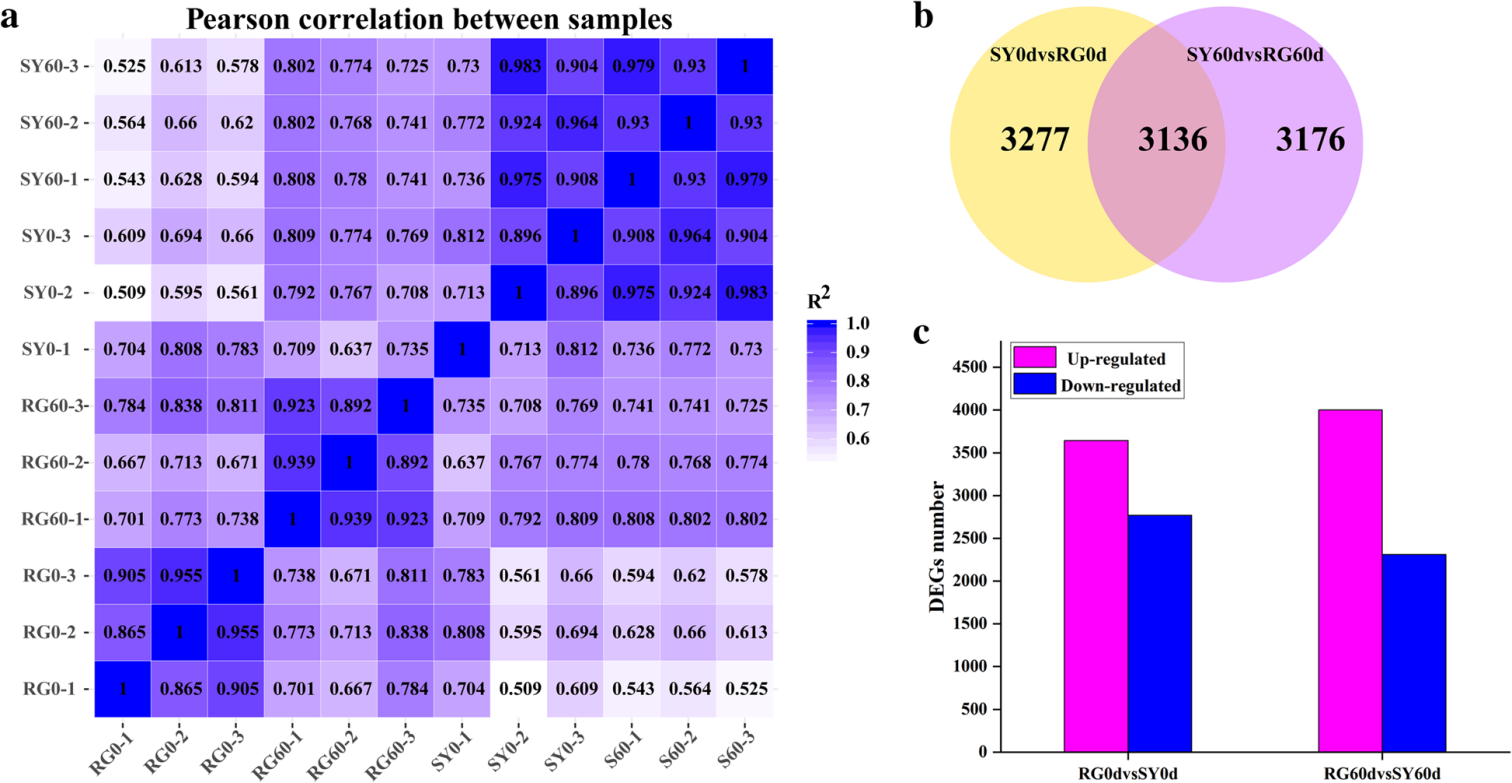
Transcriptome and differentially expressed gene analysis. **a** Pearson's correlation coefficient analysis for gene expression levels between each sample. **b** and **c** Differentially expressed genes identification both in SY0d vs. RG0d and SY60d vs. RG60d

In order to discover the candidate genes for cold resistance in *V. amurensis*, we conducted RNA-seq analysis for the dormant buds of grape varieties ‘SY’ and ‘RG’ at 0 days and 60 days after dormancy. After removing low-quality reads and adapters, a total of 77.43 Gb clean data was obtained for subsequent research, and the average clean data of each sample was 6.45 Gb. The clean data obtained by RNA-seq has been uploaded to NCBI Sequence Read Archive (SRA) with the Accession Number PRJNA787359. In total 43,047 unigenes were obtained, including 42,413 annotated genes and 634 unannotated unigenes (Supplementary file 1). These 643 unannotated unigenes were then aligned with Swiss-Prot, GO, KEGG and Pfam databases by using BLAST and HMMER software and finally annotated in Supplementary file 2. The FPKM value which was calculated by the comparison of sequenced reads with obtained RNA-seq database represents the expression of each unigene (Supplementary file 3). To confirm the reliability and rationality of the experiment, we calculated the Pearson’s correlation coefficient for all genes expression levels between each sample and refraction based on their FPKM value distribution. These coefficients are presented in the form of a correlation matrix map (Fig. 2a). A total of 6413 genes were differentially expressed in SY0d vs. RG0d (|[log2 (fold change]|) > 1 and adjusted *P* < 0.05) after differential expression analysis, among which 3644 were upregulated and 2769 were downregulated; 6312 genes were differentially expressed in SY60d vs. RG60d, among which 4002 were upregulated and 2310 were downregulated. A total of 3136 differentially expressed genes were found between SY0d vs. RG0d and SY60d vs. RG60d (Fig. 2b, c, and Supplementary file 4).

### Cold resistance genes discovery based on RNA-seq

**Fig. 3:**
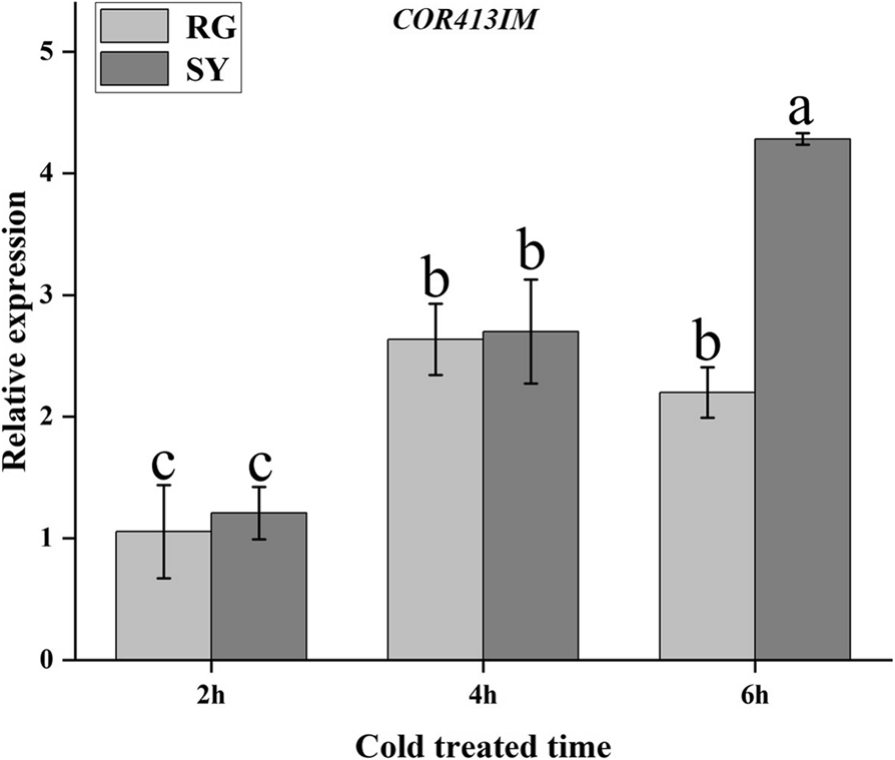
qRT-PCR analysis of cold regulated protein COR413IM at different cold treated time. Light-grey bars represent cultivar ‘RG’ and dark-grey bars represent cultivar ‘SY’. Error bars represent the standard deviation of three biological replicates. Lowercase letters on the bar chart represent significant differences between the two cultivars and different developmental stages according to Duncan’s multiple range test at *P* < 0.05

In order to cope with low temperature stress, plants produce a series of complex physiological and biochemical reactions, among which cold regulated proteins (CORs) play a crucial role in the plant response to cold stress. In this study, transcriptome analysis was conducted on the buds of ‘SY’ and ‘RG’ at different dormant periods and a cold-resistance gene, *COR413IM* (Vitvi10g00719) was discovered. The qRT-PCR analysis showed that after cold treatment, expression of *VaCOR413IM* was significantly induced and upregulated both in ‘SY’ and ‘RG’, the expression level in ‘SY’ was significantly higher than in ‘RG’ at 6 h after cold treated (Fig. 3).

**Fig. 4:**
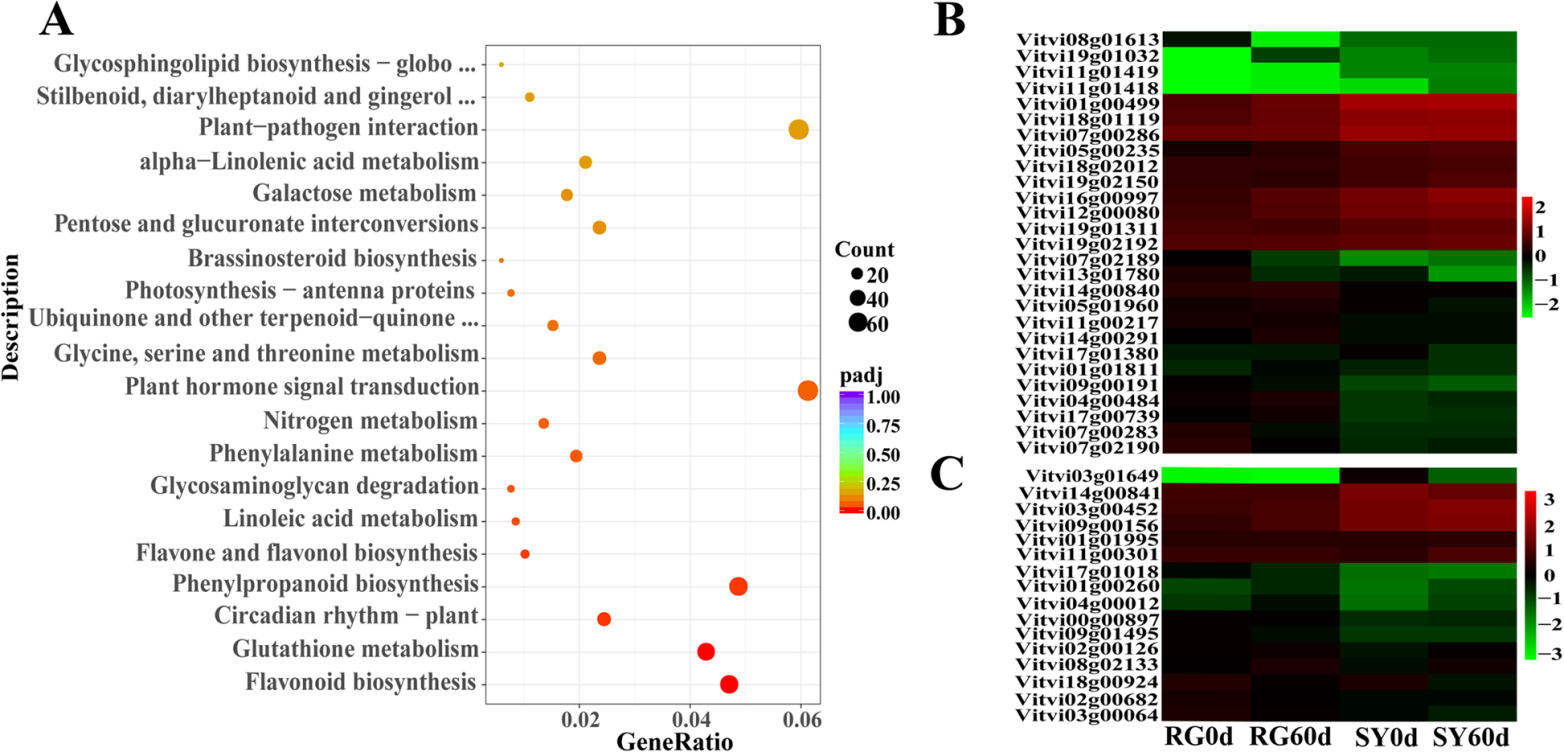
Differentially expressed genes discovery based on the transcriptome analysis of grape cultivar ‘SY’ and ‘RG’. **a** Top 20 enriched KEGG pathways related to differentially expressed genes in cultivars ‘SY’and‘RG’. The colored area represents the number of genes involved in each pathway; color intensity refers to the enrichment factor. Rich Factor defines the ratio of differentially expressed genes in the pathway; the higher is the Rich Factor, the higher is the degree of enrichment. **b** and **c** Cluster heat map of differentially expressed genes. Red corresponds to highly expressed genes and green to poorly expressed genes

**Fig. 5:**
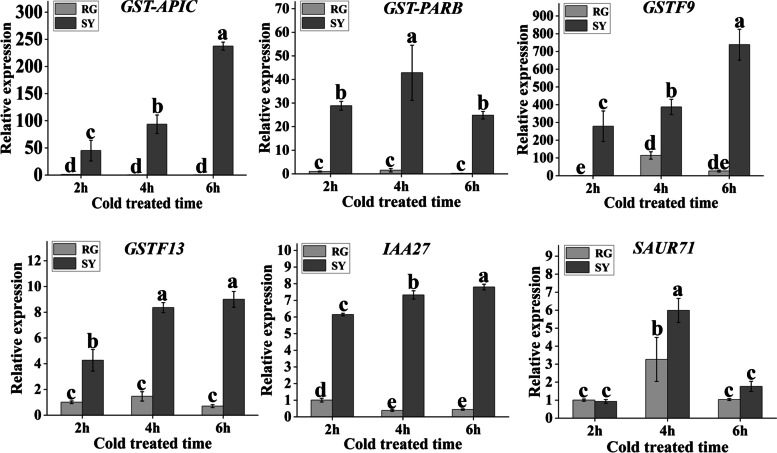
qRT-PCR analysis of candidate genes discovered based on KEGG enrichment analysis at different cold treated time. Light-grey bars represent cultivar ‘RG’ and dark-grey bars represent cultivar ‘SY’. Error bars represent the standard deviation of three biological replicates. Lowercase letters on the bar chart represent significant differences between the two cultivars and different developmental stages according to Duncan’s multiple range test at *P* < 0.05

To further explore candidate genes related to cold resistance in grapes, we conducted KEGG and GO enrichment analysis for 6312 differentially expressed genes in SY60d vs. RG60d. KEGG enrichment analysis showed that 225 genes were significantly enriched in the following pathways: flavonoid biosynthesis, glutathione metabolism, circadian rhythms-plant, phenylpropanoid biosynthesis, flavone and flavanol biosynthesis, linoleic acid metabolism, and glycosaminoglycan degradation (adjusted *P* < 0.05) (Fig. 4a). Among these, the expression levels of 28 genes in SY60d were significantly higher than those in RG60d (Fig. 4b and Supplementary file 5). Many studies have shown that hormone signal transduction plays an important role in plant cold resistance. KEGG analysis revealed that the expression of 16 genes was significantly higher in SY60d than in RG60d (Fig. 4c and Supplementary file 6). After analyzing the differential expression of selected genes in different comparison groups (RG60d vs RG0d, SY60d vs SY0d and SY0d vs RG0d) with |[log2^FC^|> 1 and adjusted *P*value < 0.05, we preliminarily screened six genes for differential expression verification (Table 1). The results showed that the expression of *GST-APIC* (Vitvi07g00283), *VaGST-PARB* (Vitvi07g02190), *VaGSTF9* (Vitvi12g00080) and *VaGSTF13*(Vitvi07g02189) in ‘SY’ were significantly upregulatedafter cold treatment, while there was almost no significant difference in ‘RG’ (*P* < 0.05); IAA27 gene in ‘SY’ was significantly upregulated after cold treatment while downregulated in ‘RG’. SAU71 was significantly induced both in ‘SY’ and ‘RG’ after cold treatment and the expression level in ‘SY’ was significantly higher than that in ‘RG’ at 4 h (Fig. 5).

**Table 1:** Cold resistance genes discovery based on RNA-seq and QTL mapping

Classification	Gene ID	Annotation	log_2_FC			
			**RG60dvsRG0d**	**SY60dvsSY0d**	**SY0dvsRG0d**	**SY60dvsRG60d**
KEGG Enrichment pathway	Vitvi07g00283	Glutathione S-transferase APIC	-4.88	0.15	-1.03	4.04
	Vitvi07g02190	Glutathione S-transferase PARB	-4.20	0.52	-1.37	3.38
	Vitvi12g00080	Glutathione S-transferase F9	0.51	0.67	2.01	2.20
	Vitvi07g02189	Glutathione S-transferase F13	-5.73	1.02	-1.66	5.11
	Vitvi11g00301	Auxin-responsive protein IAA27	-0.41	0.85	0.03	1.29
	Vitvi08g02133	Auxin-responsive protein SAUR71	1.94	0.65	2.52	1.24
GO Enrichment pathway	Vitvi09g00108	AP2-like ethylene-responsive transcription factor AIL1	-0.27	0.34	0.90	1.52
QTL Mapping& RNA-Seq	Vitvi06g00024	Myb-related protein 2	0.85	0.74	0.65	0.55
	Vitvi17g00036	Auxin response factor 2A	1.06	0.30	1.44	0.70
	Vitvi17g00037	Transcription factor ICE1	-1.63	0.04	-0.59	1.11
Transcription factor	Vitvi09g00339	Transcription factor bHLH68	-0.62	0.42	0.10	1.16
	Vitvi02g00202	bHLH transcription factor bHLH156	-0.34	0.45	0.55	1.35
	Vitvi17g00238	Myb-like protein L	0.03	0.11	1.46	1.57
	Vitvi07g00046	bHLH transcription factor bHLH059	-0.96	0.09	0.51	1.58
	Vitvi11g00320	Myb-related protein 3R-1	-2.45	0.76	-1.61	1.62
	Vitvi01g00232	Transcription factor bHLH30	-1.67	0.33	-0.01	2.01
	Vitvi14g01907	Probable WRKY transcription factor 72	-0.15	0.60	2.96	3.70

**Fig. 6:**
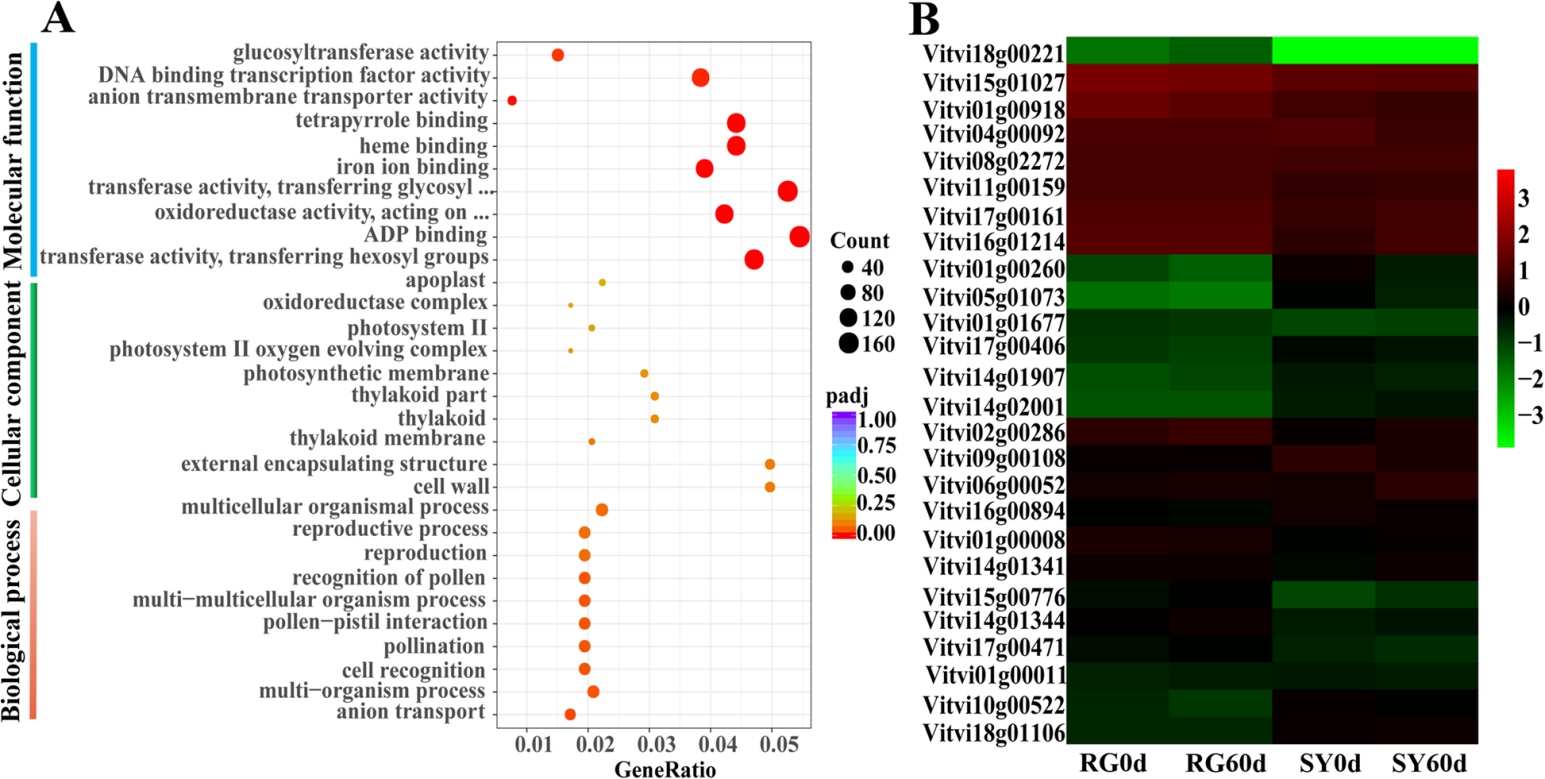
Differentially expressed genes discovery based on GO enrichment analysis for grape cultivar ‘SY’ and ‘RG’. **a** Number of different expressed genes in different catalogue. **b** Cluster heat map of differentially expressed genes. Red corresponds to highly expressed genes and green to poorly expressed genes

**Fig. 7:**
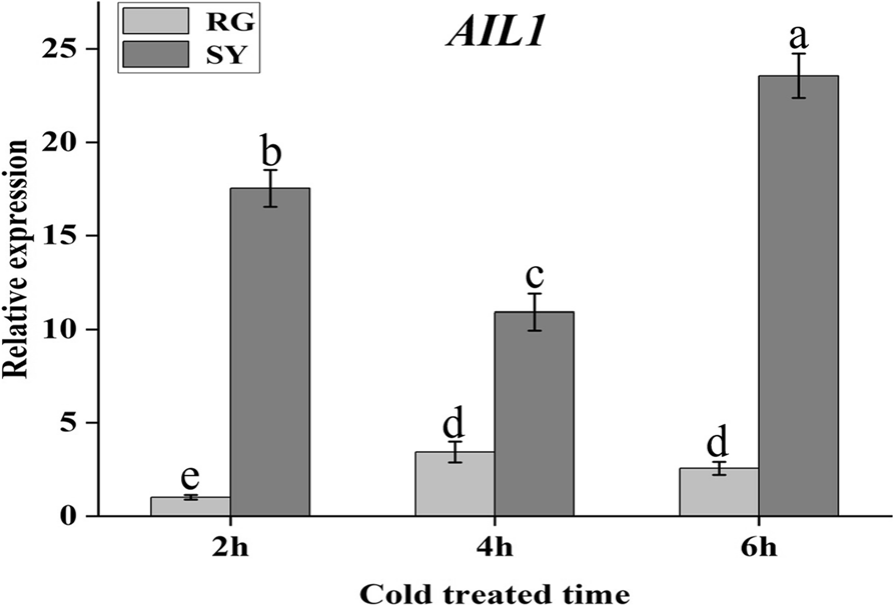
qRT-PCR analysis of ethylene response factor AIL1 at different cold treated time. Light-grey bars represent cultivar ‘RG’ and dark-grey bars represent cultivar ‘SY’. Error bars represent the standard deviation of three biological replicates. Lowercase letters on the bar chart represent significant differences between the two cultivars and different developmental stages according to Duncan’s multiple range test at *P* < 0.05

According to GO enrichment analysis, 2012 genes were significantly enriched in 30 catalogues, which were classified into categories of ‘Molecular function’, ‘Cellular component’, and ‘Biological process’ (Fig. 6a). In order to further explore candidate genes involved in transcriptional regulation mechanisms of cold resistance in grape, we focused on the analysis of genes in ‘DNA binding transcription factor activity’ catalogued under ‘Molecular function’. Finally, we identified 26 genes in which the expression level in SY60d was higher than in RG60d (Fig. 6b and Supplementary file 7). After analyzing the differential expression of selected genes in different comparison groups (RG60d vs RG0d, SY60d vs SY0d and SY0d vs RG0d), we preliminarily screened 1 gene for differential expression verification (Table 1). The results showed that ethylene response factor *AIL1* was significantly upregulated in ‘SY’ and ‘RG’, and the expression level in ‘SY’ was significantly higher than in ‘RG’ (Fig. 7).

### Cold resistance genes discovery based on QTL mapping

**Fig. 8:**
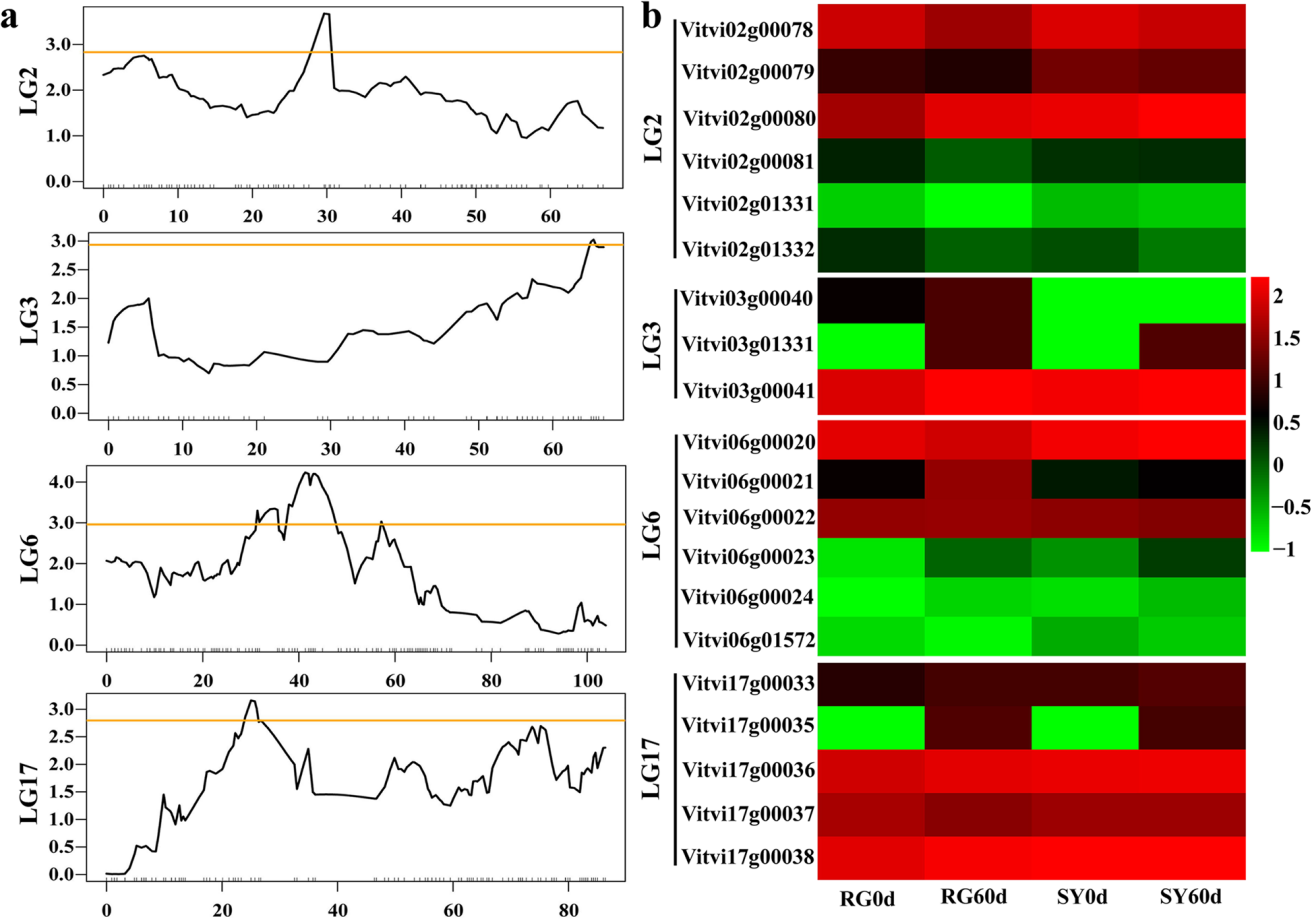
Quantitative trait locus (QTL) mapping for grape bud cold resistance. **a** The visual figure of QTL mapping in different grape linkage group. **b** Cluster heat map of differentially expressed genes in confidence intervals based on QTL mapping and transcriptome analysis. Red corresponds to highly expressed genes and green to poorly expressed genes

**Fig. 9:**
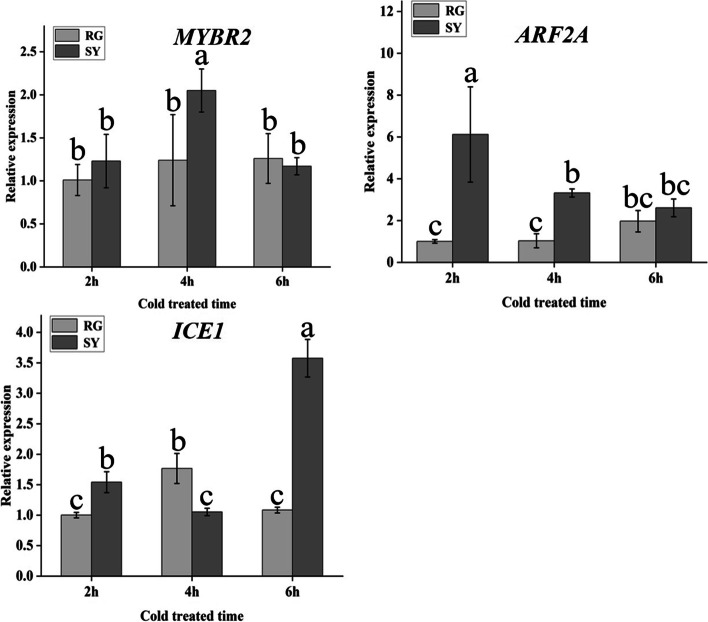
qRT-PCR analysis of candidate genes selected from QTL mapping at different cold treated time. Light-grey bars represent cultivar ‘RG’ and dark-grey bars represent cultivar ‘SY’. Error bars represent the standard deviation of three biological replicates. Lowercase letters on the bar chart represent significant differences between the two cultivars and different developmental stages according to Duncan’s multiple range test at *P* < 0.05

Based on the cold identification of hybrid offspring in 2021 and our constructed genetic linkage map, we conducted QTL mapping to further discover the candidate genes related to grape bud cold resistance. Four QTLs related to grape cold resistance were identified on LG2, LG3, LG6 and LG17, the phenotypic variation they explained ranged from 0.56% to 8.18% and the confidence intervals were 670 172–710 587, 488 381–524 863, 251 863–295 208, and 345 640–383 091, respectively (Table 2, Fig. 8a). Based on these confidence intervals, 20 candidate genes were discovered (Fig. 8b and Supplementary file 8). After analyzing the differential expression of selected genes in different comparison groups (RG60d vs. RG0d, SY60d vs. SY0d, and SY0d vs. RG0d), we preliminarily screened three genes for differential expression verification (Table 1). The results showed that the MYB transcription factor *MYBR2* was significantly upregulated in ‘SY’ while there was no significant difference in ‘RG’. The expression level in ‘SY’ was significantly higher than in ‘RG’ after cold treatment. Auxin response factor *ARF2A* was downregulated in ‘SY’ while no significant difference was seen in ‘RG’. Transcription factor *ICE1* was upregulated both in ‘SY’ and ‘RG’, the expression level in ‘SY’ was significantly higher than that in ‘RG’ (Fig. 9).

**Table 2:** Cold resistance QTL mapping based on the hybrid offspring of ‘SY’ × ‘RG’

LG	LOD threshold	Peak LOD	Peak location(cM)	PEV(%)	Confidence Interval (bp)
2	2.86	3.68	29.60	5.11	670,172–710,587
3	2.93	3.02	65.50	5.76	488,381–524,863
6	2.96	4.24	41.34	0.56	251,863–295,208
17	2.76	3.16	25.00	8.18	345,640–383,091

cM represents Centi-Morgan in linkage map and bp represents the base pair in physical map

### Transcriptional regulation factors involved in grape buds cold resistance discovery

**Fig. 10:**
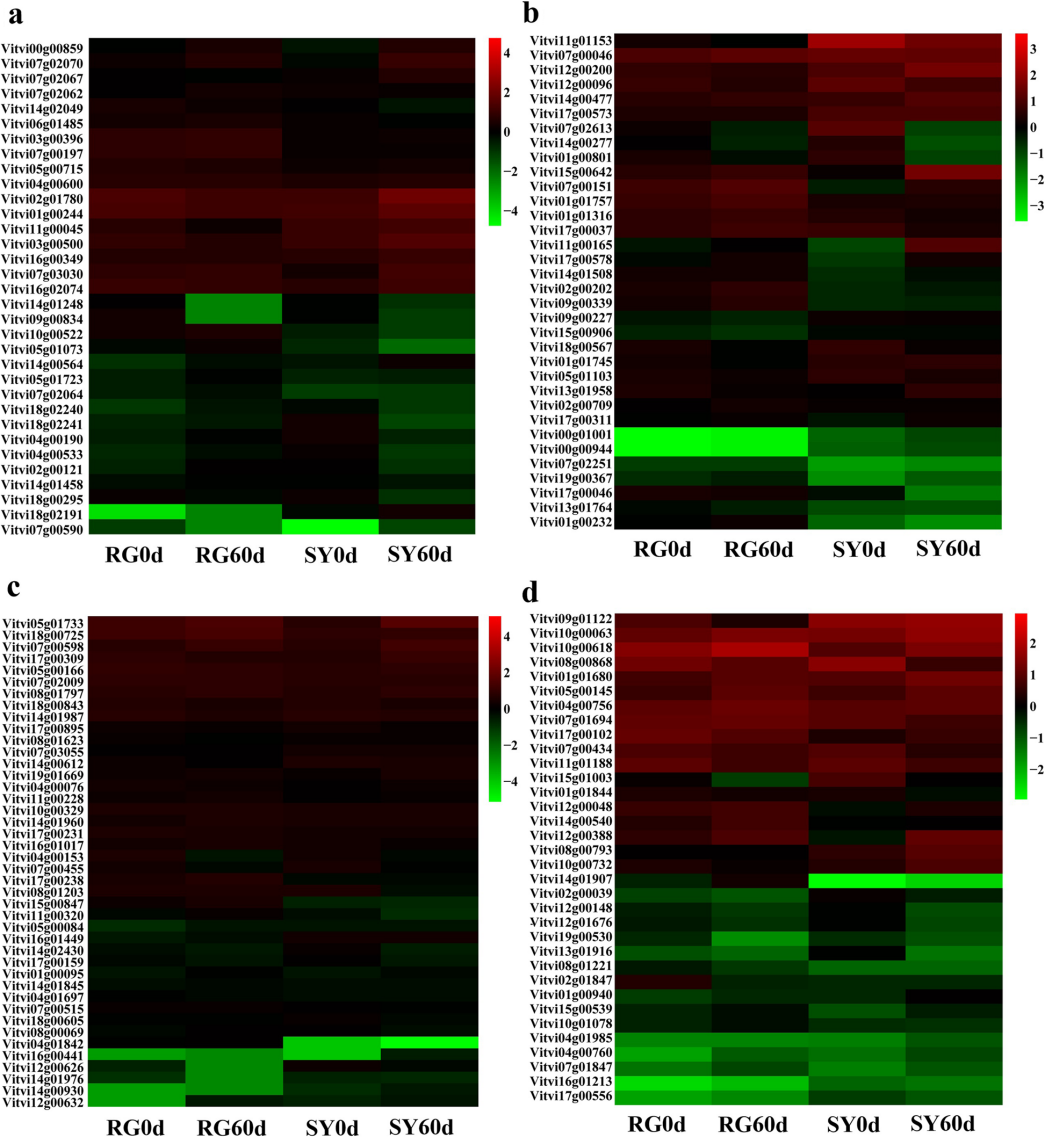
Differentially expressed genes discovery for different transcription factor family. **a** Cluster heat map of differentially expressed genes in ERF family. **b** Cluster heat map of differentially expressed genes in bHLH family. **c** Cluster heat map of differentially expressed genes in MYB family. **d** Cluster heat map of differentially expressed genes in WRKY family

**Fig. 11:**
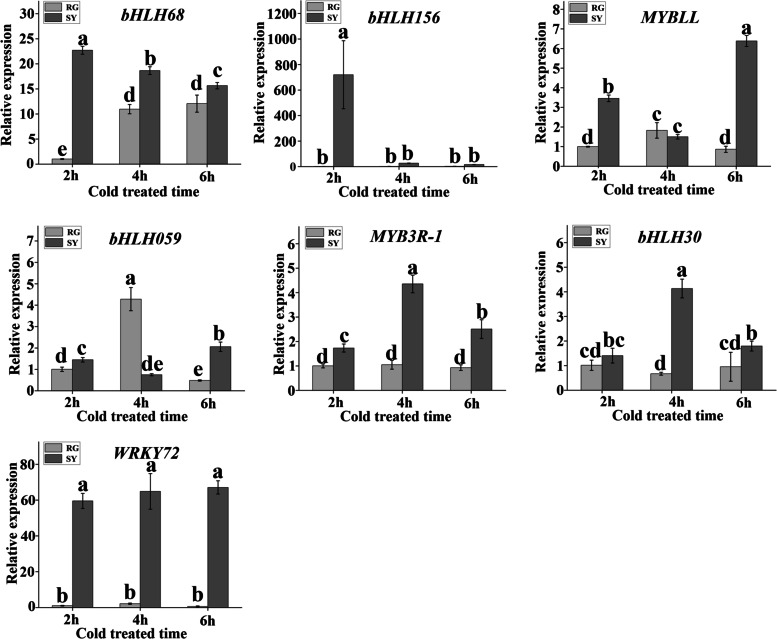
qRT-PCR analysis of transcription factor. Light-grey bars represent cultivar ‘RG’ and dark-grey bars represent cultivar ‘SY’. Error bars represent the standard deviation of three biological replicates. Lowercase letters on the bar chart represent significant differences between the two cultivars and different developmental stages according to Duncan’s multiple range test at *P* < 0.05

Additionally, we conducted analysis of ERF, bHLH, MYB, and WRKY transcription factors for their roles in resistance to environment stress. A total of 33 (ERF), 34 (bHLH), 42 (MYB) and 43 (WRKY) genes were found (Fig. 10). After analyzing the differential expression of selected genes in different comparison groups (RG60d vs. RG0d, SY60d vs. SY0d and SY0d vs. RG0d), we preliminarily screened seven genes for differential expression verification (Table 1). The results showed that the expression of WRKY72 was not different in ‘SY’ and ‘RG’ at different cold treatment stages. bHLH transcription factor bHLH68 was downregulated in ‘SY’ and upregulated in ‘RG’, and the expression level in ‘SY’ was significantly higher than in ‘RG’. bHLH156 was downregulated in ‘SY’ and there was no significant difference in the expression of ‘RG’, the expression level in ‘SY’ was significantly higher than that in ‘RG’. bHLH059 was upregulated in ‘SY’ and ‘RG’, the expression level in ‘RG’ was significantly higher than in ‘SY’. bHLH30 was upregulated in ‘SY’ and there was no significant difference in expression in ‘RG’, the expression level in ‘SY’ was significantly higher than that in ‘RG’. MYB transcription factor MYBLL was upregulated both in ‘SY’ and ‘RG’, the expression level in ‘SY’ was significantly higher than in ‘RG’. MYB3R-1 was upregulated in ‘SY’ and there was no significant difference in ‘RG’, the expression level in ‘SY’ was significantly higher than in ‘RG’ (Fig. 11).

## Discussion

In this study, some individuals showed higher cold resistance than the male parent ‘SY’. The formation of heterobeltiosis may be the additive effects of several desired dominant alleles or the combined effect of different alleles at the same gene locus, or a combination of both [16] and the genetic differences between parents are the primary cause of it. Heterobeltiosis can help breeders efficiently screen for superior parents and predict the heterosis of parental combinations [17, 18]. Transgressive offspring in our study provided important grape cold resistance resources and they can also be used as material for underlying genetic and molecular mechanisms of grape cold resistance.

Thus far, several COR genes have been identified in plants, including *COR27*, *COR47*, *COR78*, *COR15A* and *COR6*.*6* [19–28]. *COR413,* which was discovered in *Arabidopsis*, belongs to the COR subfamily and contains three proteins including COR413-plasma membrane (COR413PM), COR413-thylakoid membrane (COR413TM), and COR413-inner membrane (COR413IM) protein [29–31]. At present, COR413 proteins have been identified in rice, wheat, sorghum, and other species [29, 30, 32, 33]. As an important member of the COR gene family, no reports have been reported on the cold resistance properties of *COR413* in fruit trees and the functional research of *COR413* has mainly been focused on tomato [20, 34]. In our study, we first reported the potential cold resistance properties of *VaCOR413IM* in *V. amurensis* and have provided an important reference for future research of *COR413* in grape cold resistance. Furthermore, as a transcription factor that positively regulates COR gene expression, INDUCER OF CBF EXPRESSION (ICE1) plays an important role in plant cold resistance [35]. Previously, *VaICE1* in *V. amurensis* has been successfully cloned and its function has been verified in *Arabidopsis thaliana* and tobacco [36, 37]. In our study, we discovered a new *VaICE1* gene on LG17 in *V. amurensis* through cold-resistance QTL mapping and RNA-seq, providing a new reference for further research on the regulation of ICE1 in grape cold resistance.

Under cold stress, plants will produce a large number of reactive oxygen species (ROS) which can cause serious injury to their growth and development [38]. Glutathione S transferase (GST) can scavenge the ROS in plant tissue [39], therefore, playing an important role in improving cold resistance in plants. Multiple GST genes in pumpkin are induced by cold environments [40]. PtrERF9 improves cold resistance by promoting the expression of PtrGSTU17 and reducing ROS content in trifoliate orange under cold stress [41]. Until now, GST genes involved in grape cold resistance have not been reported. In our study, expression of four GST genes including *VaGST-APIC*, *VaGST-PARB, VaGSTF9,* and *VaGSTF13* were significantly induced and upregulated in the cold-resistant grape cultivar ‘SY’, indicating their potential role in grape cold resistance. Future studies should aim to verify their function.

Auxin response factor (ARF) and ethylene responsive factor (ERF) play important roles in plant resistance to abiotic stress [42–49]. In wheat, *TaARF8*, *TaARF9,* and *TaARF21* are involved in the response to cold stress [46]. *IAA14* in *Arabidopsis thaliana* plays an important role in mediating auxin synthesis and cold resistance [50]. Additionally, several ARF genes in sorghum are upregulated under cold stress [51]. In this study, three ARF genes *IAA27*, *SAUR71,* and *ARF2A* were discovered after QTL mapping and KEGG enrichment analysis. Expression of *IAA27* and *SAUR71* was significantly upregulated in ‘SY’ under cold stress, indicating that they could be used as candidate genes for further research into the mechanism of auxin in regulating grape cold resistance. In recent years, an increasing number of studies have reported the important regulation effect of ERF in the cold resistance of fruit trees [41, 48, 52, 53]. ERF genes involved in grape cold resistance have also been reported, including *VaERF057* [54] and *VaERF092* [5]. In this study, an ethylene response factor *VaAIL1* which may positively regulate grape cold resistance was discovered, and the specific mechanism remains to be further explored.

As the largest transcription factor family, many reports have showed the role of MYB in plant cold resistance such as: MdMYB88/124 [55], MdMYB23 [56], and MdMYB308L [57] in apple; PbrMYB5 [58] in pear; and DgMYB2 [59] in Chrysanthemum. Studies of describing the role of MYB transcription factors in grape cold resistance have been reported [7]. In this study, three MYB transcription factors *VaMYBR2*, *VaMYBLL,* and *VaMYB3R-1,* which may be involved in *V. amurensis* cold resistance were preliminarily screened through QTL mapping and transcriptome analysis, providing an important reference for further studies on MYB transcription factors involved in regulating grape cold resistance. Moreover, bHLH transcription factors MdbHLH3 [60], MdbHLH33 [57], and VabHLH1 [61]; and the WRKY transcription factors CsWRKY46 [62], SmWRKY26, and SmWRKY32 [63] play important roles in plant cold resistance. In this study, several bHLH transcription factors including bHLH68, bHLH156, bHLH059, bHLH30, and a WRKY transcription factor WRKY72 were discovered based on the transcriptome analysis of different grape cultivars under cold treatment. After qRT-PCR verification, the expression level of VabHLH30 was significantly induced and upregulated in ‘SY’, providing the possibility of bHLH30 participating in the cold resistance of *V. amurensis*. The mechanisms related to its regulations require further study.

Until now, there have been no reports of these candidate genes in relation to cold resistance. Interestingly, previous studies have shown potential interactions of these candidate genes in cold resistance. For example, the interaction between *MdMYB308L* and *MdbHLH33* could positively regulate the cold resistance in apple [57] and *PtrERF9* acts downstream of ethylene signaling and functions positively in cold tolerance via regulating *PtrGSTU17* in Citrus [41].

## Conclusions

Based on the QTL mapping and transcriptome analysis, we discovered five structural genes and eight transcription factors including *VaCOR413IM* (Vitvi10g00719), *VaGST*-*APIC* (Vitvi07g00283), *VaGST*-*PARB* (Vitvi07g02190), *VaGSTF9* (Vitvi12g00080), *VaGSTF13* (Vitvi07g02189), *VaIAA27* (Vitvi11g00301), *VaSAUR71* (Vitvi08g02133), *VaAIL1* (Vitvi09g00108), *VaMYBR2* (Vitvi06g00024), *VaMYBLL* (Vitvi17g00238), *VaMYB3R-1* (Vitvi11g00320), *VaICE1* (Vitvi17g00037) and *VabHLH30* (Vitvi01g00232). The candidate genes identified in our study will provide an important reference for research into cold-resistance mechanisms and breeding in grape species.

## Methods

### Plant material

Grape cultivar ‘Shuangyou’(SY) (*V. amurensis* Rupr.) and ‘Red Globe’(RG) (*V. vinifera* L.) and their hybrid population were cultivated in the Grape Experimental Garden of Shenyang Agricultural University (23°24’N, 41°50’E), China. In our study, October 20 in 2021 was defined as the beginning of bud dormancy, Dormant buds of ‘SY’ and ‘RG’ at 0, 30, 60 and 90 days after dormancy (DAD) were used for cold resistance identification. In order to discover candidate genes related to cold resistance, bud samples of ‘SY’ and ‘RG’ were collected at 0 DAD and 60 DAD in 2021. Three biological replicates were collected for each cultivar with at least 20 buds per replicate, the selected buds were frozen in liquid nitrogen and stored until preparation for RNA-seq analysis. The hybrid population was created in 2009 with ‘SY’ as the male parent and ‘RG’ as the female parent. Canes with buds from 149 individuals of the hybrid population were randomly collected, five canes (at least 15 buds) in each replicate were collected from three biological replicates of each individual in October 20, 2021. The canes were then placed in a refrigerator at 0 ℃ for two months to induce dormancy in the buds on the canes. After the buds were completely dormant, they were then used as materials for cold-resistance gene identification.

#### Bud cold resistance identification

Differential Thermal Analysis (DTA) was used to determine the cold resistance of dormant buds at 0DAD, 30DAD, 60DAD and 90DAD in a program-controlled cooling climate chamber (GDJS-100, Hefei Anke Environmental Testing Equipment Co., LTD., Hefei City). Full buds were removed from canes using flat cuts and then placed in a dish with moist blotting paper. The buds were placed with the flat cut downward into the thermoelectric modules (TEM) of the Tenney environmental test chamber and each module contained at least 15 buds. The methodology was as follows: temperature was decreased from room temperature to 4 °C, held at this temperature for 1 h (h); the temperature was decreased from 4 °C to -40 °C at a rate of 4 °C/h, then held at -40 °C for 1 h, and returned to room temperature over a 1 h period. The Data Loggers (CR300, Cambpell Scientific, Logan,UT) with Expansion Peripherals (AM15/32B, Cambpell Scientific, Logan, UT) is connected with the temperature sensor, scanning and collecting data every 10 s, the accuracy of which is ± 0.5℃. Meanwhile, the collected data is displayed synchronously on the computer monitor. Through the LoggerNet4.0 data collector management software (Campbell Scientific, Logan, UT), the process of the test can be observed in real time. The data is amplified and transmitted by signal amplification system and stored in Excel form to SD card. Origin2021 was used to draw the cooling curve. Cold treatment leads to an instantaneous rise in temperature and the peak value of the heat release curve is the lethal temperature of dormant buds (LTDB) [64].

#### Dormant bud transcriptome analysis

RNA integrity was assessed using the RNA Nano 6000 Assay Kit and the Bioanalyzer 2100 system (Agilent Technologies, Santa Clara, CA, USA). The input material for the RNA sample preparation was 1 μg RNA per sample. Sequencing libraries were generated using the NEBNext® Ultra™ RNA Library Prep Kit (New England Biolabs, Ipswich, MA, USA) and then sequenced on an Illumina Novaseq platform, finally, 150 bp paired-end reads were generated. Clean reads were obtained by removing reads containing adapter, ploy-N and low-quality reads from the raw data. The high quality and paired-end clean reads were aligned to the reference genome (https://www.ncbi.nlm.nih.gov/genome/401) using HISAT 2v2.0.5 software and the mapped reads of each sample were assembled by StringTie. The fragments per kilobase per million (FPKM) of each gene was calculated based on the length of the gene and the number of reads mapped to this gene. Differential expression analysis was performed using the DESeq2 R package (1.20.0) and genes with an adjusted *P*-value < 0.05 found by DESeq2 were assigned as differentially expressed. Gene Ontology (GO) enrichment analysis of differentially expressed genes was implemented by the cluster Profiler R package. Enrichment analysis of pathways in KEGG database [65–67] were performed using a hyper geometric test.

#### QTL mapping of dormant bud

The LTDB (mean value of three replicates) of each genotype collected in 2021 was used for QTL mapping. A multiple QTL mapping (MQM) method was used to find significant QTLs after a 1000-permutation test (α = 0.05) based on the R/qtl package [68]. The max.qtl was set to 10 for forward selection. A 1-LOD confidence interval corresponding to the 95% confidence interval was calculated by using the “lodint” function. The explained phenotypic variation of each QTL (PEV) was estimated using the “fitqtl” function. The candidate gene search was conducted based on the confidence interval of each QTL on the integrated map. Genes were selected according to the 12X.v3 version of the Grape Genome database (https://urgi.versailles.inra. fr/Species/Vitis/Annotations).

#### qRT-PCR validation of candidate genes

Dormant bud (60 DAD) of grape cultivars ‘SY’ and ‘RG’ which treated at -16 °C for 2 h, 4 h, and 6 h in program-controlled cooling climate chamber and then these samples were used for total RNA extraction according to the manufacturer’s instructions of Plant Total RNA Isolation Kit (SK8631; Sangon Biotech, Shanghai, China). The PrimeScript™ RT-PCR Kit (RR047A; TaKaRa Bio, Kusatsu, Japan) was used to conduct cDNA synthesis and the cDNA was diluted five times. Quantitative real-time PCR (qRT-PCR) was conducted in ABI QuantStudio™ 6 Flex System (Applied Biosystems). The relative expression level of selected genes was normalized to grapevine β-actin [69] and calculated using the 2^−ΔΔCT^ method. All reactions were performed using three biological replicates. The primers used in this study are listed in Supplementary file 9.

## Supplementary Information

**Additional file 1.** Unigenes obtained based on RNA-seq.

**Additional file 2.** Annotation analysis for unannotated unigenes.

**Additional file 3.** FPKM value of each unigene.

**Additional file 4.** Differentially expressed genes between SY60d vs. RG60d.

**Additional file 5.** The expression levels of 28 selected genes.

**Additional file 6.** Gene expression in hormone signal transduction pathway.

**Additional file 7.** Candidate genes related to cold resistance in‘DNA binding transcription factor activity’ catalogue.

**Additional file 8.** Candidate cold resistance genes selected in QTL mapping.

**Additional file 9.** The primers used in this study.

## Data Availability

The data supporting the results presented in this article are included as additional files. The raw sequencing data were deposited at the NCBI Sequence Read Archive (SRA) with the accession Number PRJNA787359.

## Abbreviations

QTL: Quantitative trait loci
LOD: Logarithm of odds
IM: Interval mapping
MQM: Multiple QTL
PGK: Phosphoglycerate kinase gene
SRA: Sequence Read Archive
CORs: Cold regulated proteins
ROS: Reactive oxygen species
GST: Glutathione S transferase
CI: Confidence interval
ARF: Auxin response factor
ERF: Ethylene responsive factor
